# Phylogenetic analyses and morphological studies reveal two new species of *Biscogniauxia* (*Xylariales*, *Ascomycota*) from Hainan Tropical Rainforest National Park, China

**DOI:** 10.3897/mycokeys.140.206566

**Published:** 2026-09-21

**Authors:** Si-Yu Ma, Shuang Liu, Zhi Qu, Huan Jiang, An-Hong Zhu, Hai-Xia Ma

**Affiliations:** 1 Haikou Key Laboratory for Conservation and Utilization of Edible and Medicinal Fungal Germplasm Resources, Hainan Key Laboratory of Tropical Microbe Resources, Institute of Tropical Bioscience and Biotechnology, Chinese Academy of Tropical Agricultural Sciences, Haikou 571101, Hainan, China Haikou Key Laboratory for Conservation and Utilization of Edible and Medicinal Fungal Germplasm Resources, Hainan Key Laboratory of Tropical Microbe Resources, Institute of Tropical Bioscience and Biotechnology, Chinese Academy of Tropical Agricultural Sciences Haikou China; 2 College of Plant Protection, Jilin Agricultural University, Changchun 130118, Jilin, China College of Plant Protection, Jilin Agricultural University Changchun China; 3 Coconut Research Institute, Chinese Academy of Tropical Agricultural Sciences, Wenchang 571339, Hainan, China Coconut Research Institute, Chinese Academy of Tropical Agricultural Sciences Wenchang China; 4 School of Ecology and Nature Conservation, Beijing Forestry University, Beijing 100083, China School of Ecology and Nature Conservation, Beijing Forestry University Beijing China; 5 Chongzuo Key Laboratory for Conservation and Utilization of Edible and Medicinal Fungal Germplasm Resources, Guangxi Research Institute, Chinese Academy of Tropical Agricultural Sciences, Chongzuo 532100, Guangxi, China Chongzuo Key Laboratory for Conservation and Utilization of Edible and Medicinal Fungal Germplasm Resources, Guangxi Research Institute, Chinese Academy of Tropical Agricultural Sciences Chongzuo China

**Keywords:** *
Graphostromataceae
*, phylogeny, species diversity, tropical rainforest, wood-inhabiting fungi

## Abstract

The species diversity of the family *Graphostromataceae* is poorly understood in tropical regions. The present work concerns a survey of wood-inhabiting macrofungi from the Hainan Tropical Rainforest, which is located in south-central Hainan Province. This study introduces two new species of *Biscogniauxia*, viz., *B. diaoluoshanensis***sp. nov**. and *B. jianfengensis***sp. nov**., which are herein described based on morphological and molecular evidence. Morphologically, *B. diaoluoshanensis* is characterized by applanate stromata, ovoid to pyriform perithecia, and ascospores with a conspicuous straight, almost spore-length germ slit. *Biscogniauxia jianfengensis* is distinguished by applanate to slightly pulvinate stromata, tubular perithecia, ascospores with an inconspicuous, unilateral, straight, spore-length germ slit, bearing an opaque cellular appendage on one side. The phylogenetic analysis inferred from ITS, *RPB2* and *TUB2* sequence data confirmed that the two new species are distinct within the genus *Biscogniauxia*. Detailed descriptions, illustrations, and phylogenetic analysis results are provided for each new species, and a dichotomous identification key is presented for the genus as well.

## Introduction

Kuntze (1891) established *Biscogniauxia* Kuntze with *B. nummularia* (Bull.) Kuntze as the type species. It is characterized by corticolous, carbonaceous bipartite stromata developing underneath the bark of the host and breaking when mature, unitunicate asci with an amyloid apical apparatus, and ellipsoid brown ascospores with germ slits and usually lacking a slimy sheath or slimy appendages (Ju et al. 1998; Fournier et al. 2017). The genus *Biscogniauxia* was resurrected and amended by Pouzar (1979, 1986) and accepted the taxa formerly placed in *Nummularia* Tul. & C. Tul. (Nom. illegit., Art. 53.1 *fide*IndexFungorum 2026). Previously, the generic concept of *Biscogniauxia* was based only on morphological characteristics and was assigned to *Xylariaceae* Tul & C. Tul (Ju et al. 1998; Vasilyeva 2007). Based on multigene phylogenetic analyses, Wendt et al. (2018) segregated the genus *Biscogniauxia* from *Xylariaceae* and transferred it to *Graphostromataceae* with *Graphostroma* Piroz., *Camillea* Fr., *Obolarina* Pouzar, and *Vivantia* J.D. Rogers, Y.M. Ju & Cand.

The genus *Biscogniauxia* is the largest genus in the *Graphostromataceae*, and has been recorded worldwide, including North America (San Martín González and Rogers 1993), Africa (Mugambi et al. 2009; Linnakoski et al. 2012), Europe (Cannon et al. 1985; Rappaz 1995; Ragazzi 2009; Ragazzi et al. 2012), Asia (Whalley et al. 2012; Jantaharn et al. 2021; Samarakoon et al. 2022; Chen et al. 2024; Qiao et al. 2024; Liu et al. 2025) and Oceania (Tan et al. 2025a, 2025b). Species of *Biscogniauxia* are widely known as saprobes, but some are plant pathogens causing diseases of dicotyledonous angiosperm trees including *Acacia*, *Acer*, *Alnus*, *Artocarpus*, *Carya*, *Celtis*, *Coprosma*, *Cydonia*, *Eucalyptus*, *Fagus*, *Fraxinus*, *Gluta*, *Lithocarpus*, *Padus*, *Phyllirea*, *Pisonia*, *Populus*, *Prunus*, *Psidium*, *Pyrus*, *Quercus*, *Rhamnus*, *Rubus*, and *Tilia* (e.g. Rappaz 1995; Ju and Rogers 1996, 2001; Rogers et al. 2000; Whalley et al. 2000; Granata and Sidoti 2004; Nugent et al. 2005; Vasilyeva 2007; Mugambi et al. 2009; Vasilyeva et al. 2012; Raimondo et al. 2016), and some are endophytes which have the potential to become pathogens when their host plants are old and stressed (Helaly et al. 2018). In addition, some species are important producers of novel bioactive compounds and secondary metabolites (Purbaya et al. 2023). Therefore, *Biscogniauxia* species are of great interest due to their ecological and economic significance.

One hundred and twenty records of *Biscogniauxia* are listed in Index Fungorum (http://www.indexfungorum.org/names/Names.asp/, accessed on 5 August 2026), and so far, more than 65 species are widely accepted (Hyde et al. 2020; Ma et al. 2020; Perera 2020; Vujanovic et al. 2020; Jantaharn et al. 2021; Samarakoon et al. 2022; Chen et al. 2024; Qiao et al. 2024; Liu et al. 2025; Tan et al. 2025a, 2025b; Ren et al. 2026; Wongkanoun et al. 2026). Among these, 17 species and 4 varieties (the formal infraspecific taxonomic rank, abbreviated as var.) have been reported and described from China, including *B. ambiens* Y.M. Ju & J.D. Rogers, *B. betulae* S.H. Long, K. Habib & Q.R. Li, *B. capnodes* (Berk.) Y.M. Ju & J.D. Rogers, *B. citriformis* (Whalley, Hammelev & Taligoola) Van der Gucht & Whalley, *B. cylindrispora* Y.M. Ju & J.D. Rogers, *B. dendrobii* X.Y. Ma, K.D. Hyde & J.C. Kang, *B. dicranopteridis* X.L. Ding, Chang Xia Qiao & D.W. Li, *B. effusa* Q.R. Li, J.C. Kang & K.D. Hyde, *B. formosana* Y.M. Ju & J.D. Rogers, *B. glaucae* Q.R. Li & J.C. Kang, *B. latirima* Y.M. Ju & J.D. Rogers, *B. petrensis* Z.F. Zhang, F. Liu & L. Cai, *B. philippinensis* (Ricker) Whalley & Læssøe, *B. plana* (Petch) Y.M. Ju & J.D. Rogers, *B. sinensis* X.L. Ding, Chang Xia Qiao & D.W. Li, *B. uniapiculata* (Penz. & Sacc.) Y.M. Ju & J.D. Rogers, *B. xishuangbannaensis* G.C. Ren & Wanas., *B. albosticta* var. *orientalis* Y.M. Ju & J.D. Rogers, *B. capnodes* (Berk.) Y.M. Ju & J.D. Rogers var. *rumpens* (Cooke) Y.M. Ju & J.D. Rogers, *B. formosana* var. *kentingensis* Y.M. Ju & J.D. Rogers, and *B. philippinensis* var. *microspora* Y.M. Ju & J.D. Rogers (Ju et al. 1998; Ju and Rogers 2001; Ariyawansa et al. 2015; Zhang et al. 2017; Ma et al. 2020; Li et al. 2021; Chen et al. 2024; Qiao et al. 2024; Liu et al. 2025; Ren et al. 2026). However, collections from tropical regions of China are few, and many undescribed novel species probably exist and are expected to be discovered. During the investigations of the diversity of wood-decaying fungi in Hainan Tropical Rainforest National Park, several specimens of *Xylariales* were collected. Two novel species of *Biscogniauxia* are identified using morphological evidence and molecular phylogenetic analyses, and a dichotomous identification key to *Biscogniauxia* taxa in China is proposed in this survey. The study makes a contribution to our understanding of the species diversity of *Graphostromataceae* in tropical China and reveals the phylogenetic relationships amongst *Biscogniauxia* species.

## Materials and methods

### Specimen collection and morphological study

The National Park of Hainan Tropical Rainforest lies in south-central Hainan Province, covering the coordinate range of 18°33'16"– 19°14'16"N and 108°44'32"– 110°04'43"E, with a tropical monsoon climate. The study area harbours 4,367 higher plant species belonging to 1,368 genera and 289 families. The dead branches bearing fungal stromata were collected from the National Park. Fresh specimens were dried, labeled, and transported for morphological and molecular studies. The studied specimens are deposited in the Fungarium of the Institute of Tropical Bioscience and Biotechnology, Chinese Academy of Tropical Agricultural Sciences (FCATAS).

The macro-morphological descriptions were observed from field notes and photos captured in the field. The stromatal surface and perithecia were observed and measured using a VHX-6000 3D microscope of the Keyence Corporation (Osaka, Japan). The micro-morphological data were obtained from dried specimens observed under an Olympus IX73 inverted fluorescence microscope (Tokyo, Japan) with a 10 × 100 oil immersion objective (Ma et al. 2022a). Sections mounted in water, Melzer’s reagent, and KOH were used to observe micro-morphological characters, following the method described by Zhu et al. (2024a). At least 30 asci and 50 ascospores from each specimen were measured randomly. The following abbreviations are used: x̄ = arithmetical average length of all ascospores × arithmetical average width of all ascospores, and n = a number of ascospores measured from a number of specimens. When presenting the variation in ascospore sizes, 5% of measurements were excluded from each end of the range and given in parentheses.

### DNA extraction, PCR amplification, and sequencing

Genomic DNA was extracted from the dried specimens using a Plant DNA Rapid Extraction Kit (Magen) according to the manufacturer’s instructions. Primer pairs used for amplification were nuclear rDNA region encompassing the internal transcribed spacer (ITS), along with the 5.8S rDNA with ITS5/ITS4 (White et al. 1990; Gardes and Bruns 1993), the portion of the β-tubulin gene (*TUB2*) with Bt2a/Bt2b (Glass and Donaldson 1995), and the RNA polymerase II subunit (*RPB2*) with RPB2-5F/RPB2-7cR (Liu et al. 1999). The PCR procedure was as follows: initial denaturation at 95 °C for 2 min, followed by 35 cycles of 95 °C for 30 s, annealing at 52 °C (ITS), 61 °C (*TUB2*), and 54 °C (*RPB2*) for 45 s each, extension at 72 °C for 1 min, and a final extension at 72 °C for 10 min. DNA sequencing was performed at Beijing Tsingke Biotechnology Co., Ltd., and all the newly generated sequences were uploaded to NCBI GenBank and listed in Table 1.

**Table 1.** List of taxa used for phylogenetic reconstruction. GenBank accession numbers, specimen numbers, host, origin, and reference studies are given. Holotype specimens are labelled with T. Species highlighted in bold were derived from this study. NA: not available.

**Specie**s	**Sample No**.	**Host**	**Locality**	**ITS**	***RPB2***	***TUB2***	**Reference**
*Biscogniauxia anceps*	YMJ 123	*Corylus avellana*	France	NA	JX507777	AY951671	Hsieh et al. 2010
*B. arima*	YMJ 122^T^	Unknown	Mexico	EF026150	GQ304736	AY951672	Crous et al. 2023
*B. atropunctata*	YMJ 128	Unknown	USA	JX507799	JX507778	AY951673	Hsieh et al. 2010
*B. australiana*	BRIP 66979a^T^	*Croton insularis*	Australia	PV074480	NA	NA	Tan et al. 2025a
*B. atropunctata* var. *intermedia*	B70M	*Quercus* sp.	Costa Rica	AJ390412	NA	NA	Sánchez-Ballesteros et al. 2000
*B. atropunctata* var. *intermedia*	ATCC 38987	Unknown	Unknown	AF201705	NA	NA	Pinto 2001
*B. bartholomaei*	ATCC 38992	Unknown	USA	AF201719	NA	NA	Pinto 2001
*B. betulae*	GMB 0711^T^	Unknown	Unknown	PQ884683	PQ893615	NA	Liu et al. 2025
*B. capnodes*	YMJ 142	*Nothofagus solandri*	New Zealand	NA	NA	AY951674	Hsieh et al. 2010
*B. capnodes*	YMJ 138	Unknown	China	NA	JX507779	AY951675	Mirabolfathy et al. 2013
*B. citriformis*	YMJ 88113012	Unknown	China	JX507800	JX507780	AY951677	Hsieh et al. 2010
*B. citriformis*	YMJ 129	Unknown	USA	JX507801	JX507781	AY951678	Mirabolfathy et al. 2013
*B. cylindrispora*	YMJ 89092701^T^	Bark of *Cinnamomum*	China	EF026133	JX507782	AY951679	Hsieh et al. 2010
*B. dendrobii*	MFLUCC 172607^T^	*Dendrobium aphyllum*	China	NR172733	NA	MK975826	Ma et al. 2020
*B. dicranopteridis*	KUNCC 23-12692^T^	*Dendrobium aphyllum*	China	OR001813	OR001855	NA	Chen et al. 2024
***B. diaoluoshanensis***	**FCATA12071^T^**	**Dead branc**h	**China**	**PX060162**	**PX092165**	**PX092167**	**This study**
***B. diaoluoshanensis***	**FCATA12659**	**Dead branch**	**China**	**PX251968**	**PX092164**	**PX092166**	**This study**
*B. formosana*	YMJ 89032201^T^	Bark	China	JX507802	JX507783	AY951680	Hsieh et al. 2010
*B. glaucae*	GMBC0007^T^	*Quercus glauca*	China	MT624046	MT622652	MT622654	Li et al. 2021
*B. glaucae*	GMBC0029	*Quercus glauca*	China	MT624047	MT622653	MT622655	Li et al. 2021
*B. granmo*	YMJ 135	Bark	Austria	JX507803	JX507784	AY951681	Hsieh et al. 2010
***B. jianfengensis***	**FCATA12140^T^**	**Dead branch**	**China**	**PX058858**	**PX092168**	**PX092170**	**This study**
***B. jianfengensis***	**FCATA12339**	**Dead branch**	**China**	**PX251984**	**PX092169**	**PX092171**	**This study**
*B. latirima*	YMJ 89101101	Bark	China	JX507804	JX507785	NA	Hsieh et al. 2010
*B. latirima*	YMJ 90080703	Bark	China	EF026135	JX507786	AY951683	Ju and Rogers 2001
*B. magna*	MFLU 18-0850^T^	Unknown	Thailand	MW240616	MW342620	MW775577	Samarakoon et al. 2022
*B. mangiferae*	MFLU 18-0827^T^	*Mangifera indica*	Thailand	MN337232	MN366247	MN509782	Samarakoon et al. 2022
*B. marginata*	MFLUCC 12-0740	Unknown	France	KJ958407	KJ958409	KJ958406	Daranagama et al. 2015
*B. marginata*	CBS 124505	*Quercus* sp.	USA	KU684016	KU684310	KU6841249	Chen et al. 2024
*B. mediterranea*	YMJ 147^T^	Unknown	France	EF026134	NA	AY951684	Hsieh et al. 2010
*B. mediterranea*	AZ0703	Unknown	USA	HM123416	KU684217	KU684122	U’Ren et al. 2016
*B. nummularia*	MUCL 51395^T^	*Fagus sylvatica*	France	NR 153649	NA	KX271241	Wendt et al. 2018
*B. nummularia*	CBS 969.70	Unknown	Unknown	MH860015	KU684281	KU684125	Li et al. 2021
*B. papillata*	BCC 36828^T^	Unknown	Unknown	PQ586363	PQ604605	NA	Wongkanoun et al. 2026
*B. petrensis*	LC5698	Unknown	China	KU746670	NA	KU746763	Blumenstein et al. 2021
*B. petrensis*	MFLUCC 14-0151	*Dendrobium harveyanum*	Thailand	MK944283	MK975825	MK975827	Ma et al. 2020
*B. petrensis*	HKAS 102388^T^	Unknown	Unknown	MW240615	MW342619	MW775576	Hsieh et al. 2010
*B. philippinensis* var. *microspora*	YMJ 89041101	Bark	China	EF026136	JX507787	AY951685	Hsieh et al. 2010
*B. pezizoides*	ATCC 62606	Unknown	USA	KY610383	KY624237	KX271242	Brenken and Riebesehl 2025
*B. repanda*	KAS-JKI-GF-2023-009	Unknown	Czech Republic	PQ227787	NA	NA	Brenken and Riebesehl 2025
*B. rosacearum*	Bx63	*Quercus pubescens*	Italy	KT253501	NA	KT253535	Raimondo et al. 2016
*B. rosacearum*	Bx26^T^	*Prunus domestica*	Italy	KT253493	NA	KT253527	Raimondo et al. 2016
*B. sinensis*	SC3^T^	*Pinus thunbergii*	China	OR803755	OR832189	OR832179	Qiao et al. 2024
*B. simplicior*	YMJ 136	*Rhamnus cathartica*	France	EF026130	JX507788	AY951686	Hsieh et al. 2010
*B. uniapiculata*	YMJ 90080608	Bark	China	JX507805	JX507789	AY951687	Hsieh et al. 2010
*B. xishuangbannaensis*	HKAS 122774	Dead twig	China	PX583847	PX521740	NA	Ren et al. 2026
*Camillea broomeana*	GMBC0218	Unknown.	China	MW854657	MW855488	MW855491	Li et al. 2021
*C. obularia*	ATCC 28093	Unknown	USA	KY610384	KY624238	KX271243	Wendt et al. 2018
*C. tinctor*	MFLU 18-0786	Unknown	Unknown	MW240614	MW342618	MW775575	Samarakoon et al. 2022
*C. tinctor*	YMJ 363	Unknown	Martinique	JX507806	JX507790	JX507795	Hsieh et al. 2010
*Cryptostroma corticale*	CBS 216.52	*Acer* sp.	United Kingdom	HG934110	HG934116	HG934102	Li et al. 2021
*Cr. corticale*	CBS 218.52	*Acer* sp.	United Kingdom	HG934112	HG934118	HG934104	Li et al. 2021
*Graphostroma guizhouensis*	GMBC0008	Unknown	China	MW854658	MW855486	MW855489	Li et al. 2021
*G. guizhouensis*	GMBC0219^T^	Unknown	China	MW854659	MW855487	MW855490	Li et al. 2021
*G. platystomum*	CBS.270.87^T^	Unknown	France	HG934115	KY624296	HG934108	Li et al. 2021
*G. platystomum*	CPC:37153	*Lindera benzoin*	USA	GQ428316	NA	NA	Li et al. 2021
*Hypoxylon rubiginosum*	YMJ 24	*Quercus brantii*	Iran	EF026143	JX507791	AY951751	Hsieh et al. 2010
*Jackrogersella cohaerens*	YMJ 310	*Fagus* sp.	Unknown	EF026140	GQ844766	AY951655	Hsieh et al. 2010
*Obolarina dryophila*	8409	Unknown	Unknown	GQ428316	NA	GQ428321	Li et al. 2021
*O. dryophila*	H76	*Quercus* sp.	France	GQ428317	NA	GQ428323	Li et al. 2021
*O. persica*	YMJ 1461^T^	Unknown	Unknown	JX507807	JX507792	JX507796	Li et al. 2021

### Phylogenetic analyses

Phylogenetic analyses for *Biscogniauxia* and related genera, including *Graphostroma*, *Camillea*, *Cryptostroma*, and *Obolarina*, were performed with Bayesian inference (BI) and Maximum likelihood (ML) analyses based on the combined ITS-*RPB2*-*TUB2* dataset (Table 1). *Jackrogersella cohaerens* (Pers.) L. Wendt, Kuhnert & M. Stadler and *Hypoxylon rubiginosum* (Pers.) Fr. were selected as outgroups (Stadler et al. 2013; Wendt et al. 2018; Li et al. 2021; Crous et al. 2023). The sequences were aligned using the online analysis website MAFFT (MAFFT alignment and NJ/UPGMA phylogeny). The aligned sequences were trimmed using BioEdit software (Hall 1999), and individual gene datasets were concatenated using MEGA 6.0 (Tamura et al. 2011). The maximum likelihood (ML) phylogenetic tree was constructed using the RAxML GUI v1.3.1 software, employing the GTRGAMMA model and the ML+rapid bootstrap procedure with 1,000 replicates to assess the confidence of each branch (Stamatakis et al. 2008). The trimmed sequences were converted to NEXUS format, and manual adjustments were made using ClustalX 1.83 (Thompson et al. 1997). Bayesian inference (BI) analyses were performed using MrBayes v.3.2.6 (Ronquist et al. 2012). The best-fit substitution models for each gene were determined using jModelTest 2 (Darriba et al. 2012). Six Markov chains were run for 3,000,000 generations, with trees sampled every 100 generations. The first 25% of the sampled trees were discarded as burn-in, and the remaining trees were used to calculate posterior probabilities (Rannala and Yang 1996). The phylogenetic tree was visually edited using FigTree v1.4.4 (http://tree.bio.ed.ac.uk/software/figtree/).

## Results

### Phylogenetic inference

The phylogenetic analysis was based on a total of 148 sequences from three DNA loci (56 ITS, 44 *RPB2*, and 48 *TUB2*) derived from 61 strains representing 43 species, including two newly described species, 31 known species of *Biscogniauxia*, 3 species of *Camillea*, one species of *Cryptostroma*, 2 species of *Graphostroma*, 2 species of *Obolarina*, and the outgroups *Jackrogersella cohaerens* (YMJ 310) and *Hypoxylon rubiginosum* (YMJ 24).

**Figure 1.**
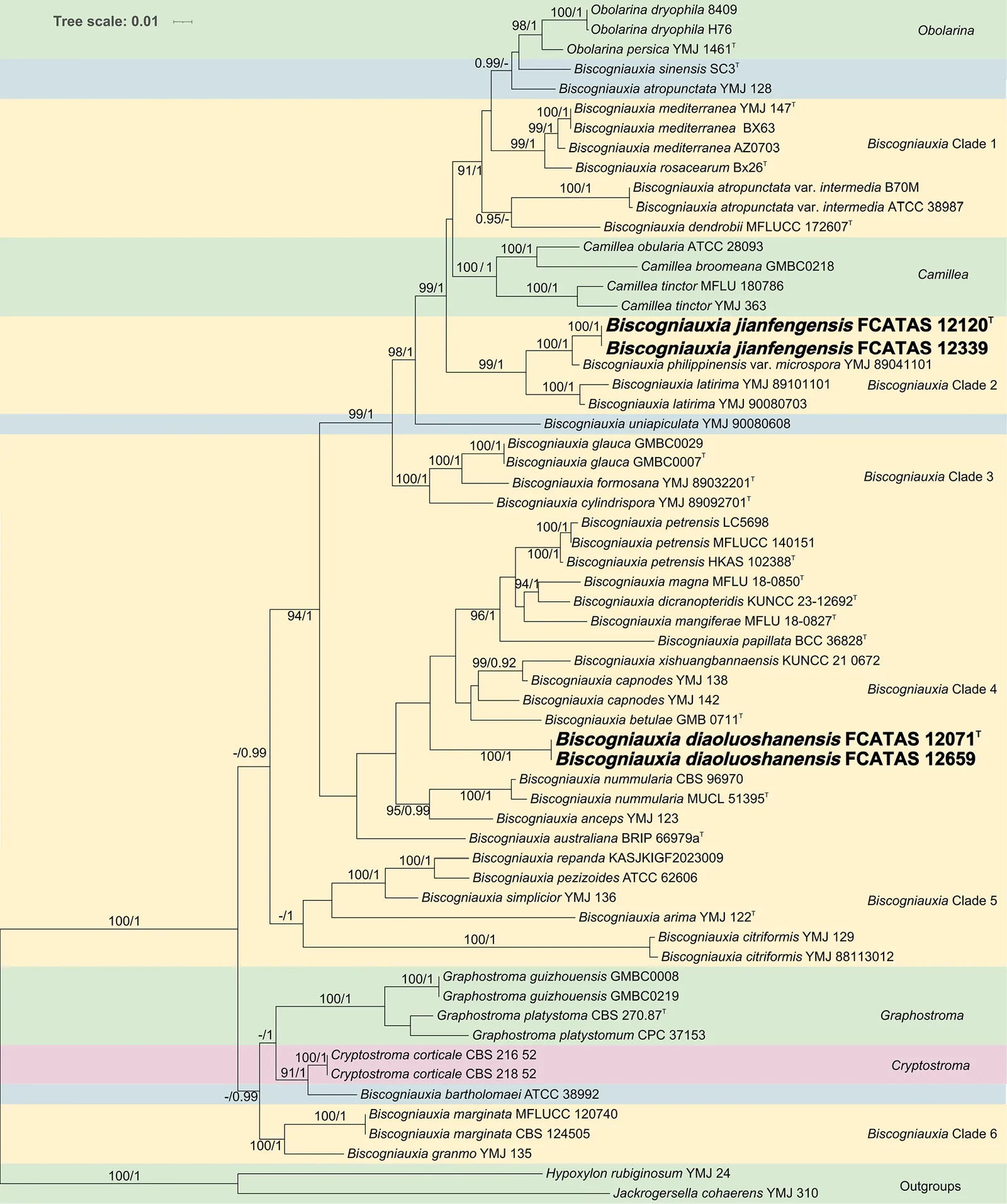
Phylogram generated from Maximum Likelihood (RAxML) analyses based on ITS-*RPB2*-*TUB2* matrix. Branches are labelled with maximum likelihood bootstrap ≥80% and Bayesian posterior probabilities ≥0.90 at the nodes. New species in this study are indicated in bold. Holotype specimens are labelled with T.

The dataset contained a total of 3,233 aligned characters, of which 1,683 were constant characters, 1,326 were phylogenetically informative characters, and 224 were variable characters that lacked parsimony information. Phylogenetic trees generated from ML and BI analyses of the combined dataset of ITS-*RPB2*-*TUB2* were highly similar in topology. Only the ML tree is represented in Fig. 1, with ML bootstrap values ≥ 80% and Bayesian posterior probabilities ≥0.90 labelled along the branches. The phylogram based on the combined ITS-*RPB2*-*TUB2* sequences (Fig. 1) showed that our two species were assigned to the genus *Biscogniauxia*, in which *B. jianfengensis* grouped with *B. philippinensis* var. *microspora* Y.M. Ju & J.D. Rogers (YMJ 89041101) with high support value (BS = 100, BPP = 1), and *B. diaoluoshanensis* resolved as a basal lineage of the other *Biscogniauxia* taxa included in this study. Based on phylogenetic and morphological evidence (described below), we propose two new species, namely *B. diaoluoshanensis* sp. nov. and *B. jianfengensis* sp. nov., to accommodate our specimens.

### Taxonomy

#### Biscogniauxia diaoluoshanensis

Taxon classificationFungiXylarialesGraphostromataceae

Hai X. Ma, A.H. Zhu, S. Liu & S.Y. Ma sp. nov.

0C65A224-C1F5-5013-8AAC-835D471D14A1

864450

Fig. 2

##### Holotype.

China • Hainan Province, Lingshui Li Autonomous County, Hainan Tropical Rainforest National Park, Diaoluo Mountain Branch, Fengguoshan Waterfall, saprobic on a dead branch of an unidentified plant, alt. 530 m, 27 May 2024, Hai X. Ma, DLS058 (FCATAS 12071, Holotype). GenBank accession numbers: ITS (PX060162); *TUB2* (PX092167); *RPB2* (PX092165).

**Figure 2.**
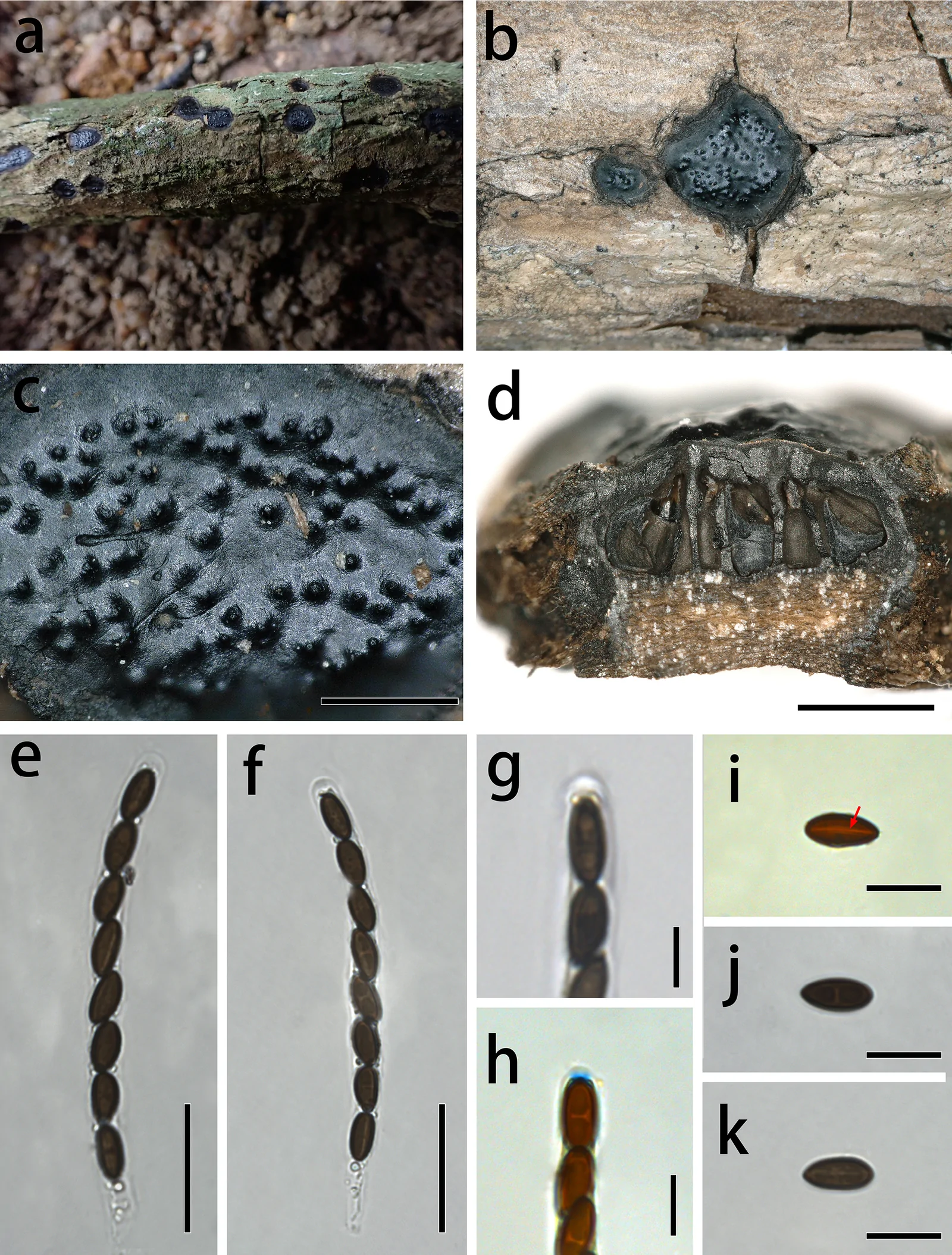
*Biscogniauxia diaoluoshanensis*. (FCATAS 12071, Holotype). **a, b**. Stromata on the surface of the branch; **c**. Surface of stromata; **d**. Vertical section through stroma showing ostioles and perithecia; **e, f**. Asci in water; **g**. Apical ring of the asci; **h**. Ascal ring bluing in Melzer’s reagent; **i**. Ascospore with germ slit; **j**. Ascospore in water; **k**. Ascospore in KOH. Scale bars: 0.5 mm (**c, d**); 25 μm (**e, f**); 10 μm (**g–k**).

##### Etymology.

Named after Diaoluo Mountain Branch of Hainan Tropical Rainforest National Park, the type locality in Hainan Province, China.

##### Description.

**Sexual morph: *Stromata*** applanate, orbicular to irregularly lobed, 4–12 × 1.5–5 cm, with slightly sloping margins, somewhat confluent; mature surface black, shiny, with conspicuous depressions, tissue beneath perithecia 0.03–0.04 mm thick, carbonaceous, underlying bark tissue black. ***Perithecia*** ovoid to pyriform, frequently laterally and basally flattened, 340–430 × 70–115 µm (x̄ = 385 × 92 µm, n = 20), opening centrally or laterally through individual ostioles. ***Ostioles*** papillate, apically obtuse or truncate, black, conspicuously shiny, at times punctate with a low rim. ***Asci*** cylindrical with 8 ascospores, 89–95 µm total length, short-stipitate to subsessile, the spore-bearing parts 76–80 × 6–8 µm (x̄ = 78 × 6 µm, n = 60/2), the stipes 12–14 µm; apical apparatus 2.6–5.7 × 5.3–5.7 µm (x̄ = 1.7 × 5.1 µm, n = 60/2), short-cylindrical, slightly trapezoid, bluing in Melzer’s reagent (J+). ***Paraphyses*** sparse, hyphal, unbranched, thin-walled, septate, 4–5 µm wide, tapering above asci. ***Ascospores*** (10.1–) 10.4–12.7 (–13.1) × (4.8–) 5.1–6.1(–6.3) µm (x̄ = 11.6 × 5.5 µm, n = 60), unicellular, light brown to brown, ellipsoid almost equilateral, one end slightly broader and rounded, the other slightly narrower and obliquely truncate, smooth-walled, with a conspicuous straight germ slit almost spore-length or slightly less than spore-length. **Asexual morph**: Undetermined.

##### Additional specimens examined.

China • Hainan Province, Lingshui Li Autonomous County, Hainan Tropical Rainforest National Park, Diaoluo Mountain Branch, Fengguoshan Waterfall, saprobic on a dead branch of an unidentified plant, alt. 527 m, 27 May 2024, Hai X. Ma, DLS061 (FCATAS 12659, Paratype).

##### Remarks.

*Biscogniauxia diaoluoshanensis* is characterized by slightly concave stromata with a blackish-brown surface and shiny black papillate ostioles, and nearly equilateral brown ascospores with a conspicuous straight, almost spore-length germ slit. *Biscogniauxia granmoi* Lar. N. Vassiljeva resembles the new species in its stromatal morphology and ascospore shape but possesses larger perithecia (0.6–0.8 mm high × 0.3–0.5 mm broad) and larger asci (119–142 µm total length), and occurs on trunks and branches of *Prunus* (Vasilyeva 1998; Læssøe et al. 1999). Another similar species, *B. nummularia* (Bull.: Fr.) Kuntze, resembles the new species in stromatal appearance, but can be distinguished by its larger obovoid perithecia (0.3–0.5 × 0.5–0.7 mm), longer asci (95–120 µm total length), and wider ascospores (7.5–8.5 µm) (Ju et al. 1998; Ju and Rogers 2001). In addition, it occurs on wood of *Fagus* (Ju et al. 1998; Zíbarová and Kout 2017). Phylogenetically, the new species falls within *Biscogniauxia* clade 4 yet exhibits no obvious affinity to other taxa in this clade. Clade 4 comprises the type species *B. nummularia*, ten additional *Biscogniauxia* species, and our new collection. *Biscogniauxia nummularia* (MUCL 51395, Holotype) differs from *B. diaoluoshanensis* by 168 base pairs in *TUB2* alignment, representing 11.52% of the total aligned *TUB2* region. Strain CBS 969.70 of *B. nummularia* shows 95-base-pair divergence in *RPB2* and 164-base-pair divergence in *TUB2* compared with the new species, corresponding to 9.27% and 11.28% of aligned nucleotide positions of *RPB2* and *TUB2* regions, respectively. *Biscogniauxia capnodes* (YMJ 138) differs from *B. diaoluoshanensis* by 83 base pairs in *RPB2*, accounting for 7.57% of the aligned *RPB2* region. For other congeneric species, sequence similarity to the new species is below 91% for both *RPB2* and *TUB2*. All previously known species display ITS sequence similarity below 86% relative to this novel taxon.

#### Biscogniauxia jianfengensis

Taxon classificationFungiXylarialesGraphostromataceae

Hai X. Ma, A.H. Zhu, S.Y. Ma & S. Liu sp. nov.

6C64DE9F-A69F-5118-BE2B-B7C1AD4E7340

864451

Fig. 3

##### Holotype.

China • Hainan Province, Ledong Li Autonomous County, Hainan Tropical Rainforest National Park, Jianfengling Branch, Yulin Valley, alt. 650 m, saprobic on a dead branch of an unidentified plant, 29 May 2024, Hai X. Ma, JFL0041 (FCATAS 12140, Holotype). GenBank accession numbers: ITS (PX058858); *TUB2* (PX092170); *RPB2* (PX092168).

**Figure 3.**
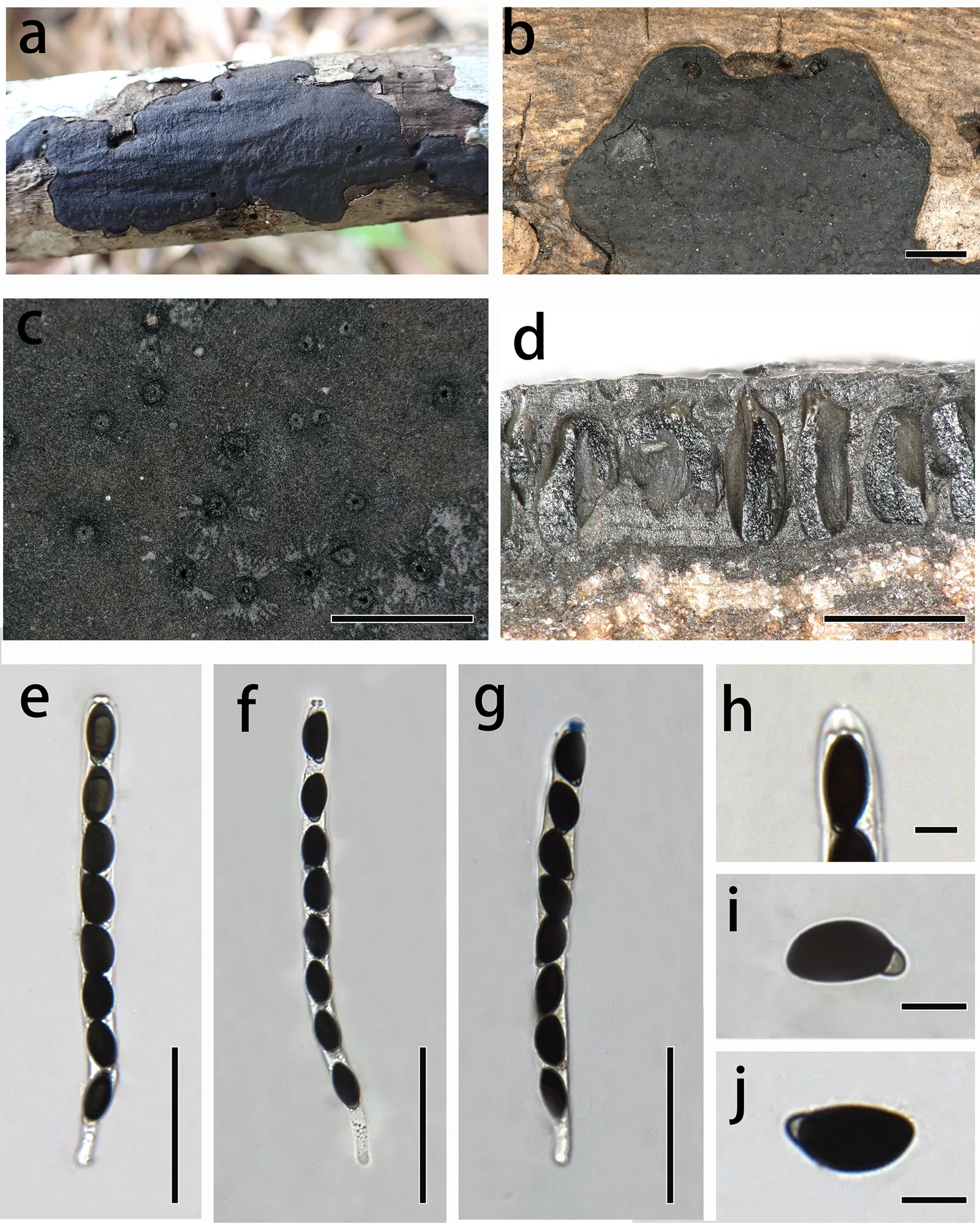
*Biscogniauxia jianfengensis* (FCATAS 12140, Holotype). **a, b**. Stromata on the surface of the branch; **c**. Surface of stromata; **d**. Vertical section through stroma showing ostioles and perithecia; **e, f**. Asci in water; **g**. Ascal ring bluing in Melzer’s reagent; **h**. Apical ring of the asci in water; **i, j**. Ascospore in water. Scale bars: 1 mm (**b**); 0.5 mm (**c, d**); 50 μm (**e–g**); 10 μm (**h–j**).

##### Etymology.

Named after Jianfengling Branch of Hainan Tropical Rainforest National Park, the type locality in Hainan Province, China.

##### Description.

**Sexual morph: *Stromata*** applanate to slightly pulvinate, orbicular to irregularly ellipsoid-lobed, 2–6 × 1–3 cm, coalescent into widely effused compound stromata, with slightly sloping margins lacking remnants of the outer dehiscing layer; mature stromatal surface dull black, matte, even, and carbonaceous immediately beneath the surface and between perithecia; tissue beneath perithecia 0.05–0.07 mm thick, carbonaceous, underlying bark tissue black. ***Perithecia*** tubular to short-tubular, 450–570 × 210–480 µm (x̄ = 506 × 289 µm, n = 20), opening centrally or laterally through individual ostioles. ***Ostiole*** umbilicate or punctate with a slightly to conspicuously raised rim, often surrounded by a darker halo, evenly distributed or in small clusters. ***Asci*** cylindrical with 8 ascospores, 125–186 µm total length, short-stipitate to subsessile, the spore-bearing parts 121–148 × 9–11 µm (x̄ = 136 × 10 µm, n = 60/2), the stipes 10–25 µm; apical apparatus 2.6–5.7 × 6.6–9.2 µm (x̄ = 4.1 × 7.6 µm, n = 60/2), short-cylindrical, apically flared, bluing in Melzer’s reagent (J+). ***Paraphyses*** copious, hyphal, unbranched, thin-walled, septate, 4–6 µm wide, tapering above asci. **Ascospores** (13.4–) 14.8–18.8 (–20.8) × (8.3–) 8.5–10.6 (–11.4) μm (x̄ = 17.1 × 9.6 μm, n = 50), unicellular, dark brown to blackish brown, ellipsoid-inequilateral with broadly rounded upper end, bearing an opaque, thick-walled broadly conical, obtusely-ended cellular appendage, 1.6–2.8 µm high × 2.3–3.8 µm broad (x̄ = 2.2 × 3.2 µm, n = 30), with an inconspicuous, unilateral, straight spore-length or slightly less germ slit on the most convex side. **Asexual morph**: Undetermined.

##### Additional specimens examined.

China • Hainan Province, Ledong Li Autonomous County, Hainan Tropical Rainforest National Park, Jianfengling Branch, alt. 660 m, saprobic on a dead branch of an unidentified plant, 29 May 2024, Hai X. Ma, (FCATAS 12339, Paratype). GenBank accession numbers: ITS (PX251984); *TUB2* (PX092171); *RPB2* (PX092169).

##### Remarks.

Phylogenetically, *Biscogniauxia jianfengensis* forms a sister lineage to the tropical variety *B. philippinensis* var. *microspore*, with this clade itself sister to *B. latirima* Y.M. Ju & J.D. Rogers. *Biscogniauxia philippinensis* var. *microspore* resembles the new species in stromatal morphology and ascospore shape but differs in having a larger perithecia (1.4–2.4 × 0.2–0.3 mm vs. 450–570 × 210–480 µm) and longer asci with the spore-bearing parts (asci 190–220 µm total length vs. 125–186 µm total length) (Ju et al. 1998). In contrast, *B. latirima* shares applanate stromata and similar perithecia shape but diverges in smaller apical apparatus (4–5 × 5–6 µm vs. 2.6–5.7 × 6.6–9.2 µm), and larger ascospores (18–21.5 × 10–12 µm vs. 14.8–18.8 × 8.5–10.6 μm) lacking a visible cellular appendage (Ju and Rogers 2001). Morphologically, the new species *B. jianfengensis* is similar to the tropical taxon *B. philippinensis* in stromatal morphology, but the latter has larger ascospores (22–27 × 11.5–13 µm) with a straight, faint spore-length germ slit (Ju et al. 1998). Unfortunately, no molecular sequences are available for *B. philippinensis*, so nucleotide difference comparison could not be implemented.

## Discussion

In the present study, two new species of *Biscogniauxia* from Hainan Tropical Rainforest National Park in China are described based on morphological characteristics and phylogenetic analyses. Phylogenetic analyses on the species of *Biscogniauxia* presented confirmed that the genus *Biscogniauxia* is polyphyletic, as in previous studies (Ma et al. 2020; Li et al. 2021; Chen et al. 2024; Liu et al. 2025; Liu et al. 2026). The species analyzed appeared mainly distributed in six separate clades (except *B. sinensis*, *B. atropunctata* (Schwein.) Pouzar, *B. uniapiculata*, and *B. bartholomaei* (Peck) Lar.N. Vassiljeva) in our study. *Biscogniauxia diaoluoshanensis* and *B. jianfengensis* were clearly separated from other *Biscogniauxia* species and from each other in *Biscogniauxia* clade 4 and clade 2 respectively. *Biscogniauxia nummularia*, the type species of the genus, was included in clade 4. The phylogenetic tree indicates that the classification status of the genus *Biscogniauxia* remains ambiguous. In our phylogenetic analyses, *Camillea* species nested within the *Biscogniauxia* clade, which is consistent with recent findings reported by Wongkanoun et al. (2026). Accordingly, further studies on the diversity, phylogeny, and evolution of *Biscogniauxia* require expanded sampling and molecular data from broader geographic regions.

A number of macrofungi belonging to *Basidiomycota* and *Ascomycota* have been extensively studied in recent years, and numerous new taxa have been described from Hainan Province (Ma et al. 2022b; Pan et al. 2022; Deng et al. 2023, 2024; Huang et al. 2023; Gao et al. 2024; Zhu et al. 2024b; Yu et al. 2025; Zhang et al. 2025; Li et al. 2025). Members of *Graphostromataceae* are widely distributed across tropical regions worldwide; nevertheless, records from tropical Asia remain relatively sparse (Samarakoon et al. 2022; Liu et al. 2025; Ren et al. 2026; Wongkanoun et al. 2026). To date, only three species of *Graphostromataceae* have been documented from Hainan Province (Ju et al. 1998; Ariyawansa et al. 2015). Therefore, the species diversity of *Graphostromataceae* is still largely underexplored on this tropical island, and further surveys across diverse forest types in Hainan are likely to yield additional new species.

### Dichotomous key to *Biscogniauxia* taxa known from China

Note that taxa with only anamorph reported are not included in this key.

**Table:** 

1	Mature ascospores bearing a cellular appendage	**2**
–	Mature ascospores lacking a cellular appendage	**5**
2	Ascospores with a conspicuous straight germ slit, (9–)10–14 × 5–7 µm	***B. uniapiculata***
–	Ascospores with an unconspicuous germ slit	**3**
3	Ascospores 22–27 × 11.5–13 µm	***B. philippinensis***
–	Ascospores smaller than 21 µm	**4**
4	Perithecia 0.45–0.57 mm high × 0.21–0.48 mm broad; ascospores (13.4–) 14.8–18.8 (–20.8) × (8.3–) 8.5–10.6 (–11.4) μm	***B. jianfengensis* sp. nov**.
–	Perithecia 1.4–2.4 mm high × 0.2–0.3 mm broad; ascospores 13.5–19 × 8–10 µm	***B. philippinensis* var. *microspora***
5	Ascospores inequilateral to strongly inequilateral	**6**
–	Ascospores more or less equilateral	**9**
6	Ascospores 8.5–15 × 5–7.5 μm	***B. citriformis***
–	Ascospores greater than 15 μm long	**7**
7	Ascospores (16–)18–21.5 × 10–12 μm, with a broad germ-slit on more convex side	***B. latirima***
–	Ascospores with a narrow germ-slit on less convex side	**8**
8	Ascospores dark brown, (18–)19–24(–25) × 8.5–10 μm	***B. formosana***
–	Ascospores pale brown, narrow, 17–23.5 × 10–12 μm	***B. formosana* var. *kentingensis***
9	Ascospores light brown to brown	**10**
–	Ascospores dark brown to blackish brown	**11**
10	Ascospores (10.1–) 10.4–12.7 (–13.1) × (4.8–) 5.1–6.1(–6.3) µm	***B. diaoluoshanensis* sp. nov**.
–	Ascospores 25–31 × 8–10 µm	***B. effusa***
11	Stromata widely effused, with raised margins	**12**
–	Stromata applanate	**13**
12	Ascospores 13.5–16 × 7.5–9.5 μm	***B. glaucae***
–	Ascospores 10–12.5 × 6.5–10 μm	***B. betulae***
13	Mature stromatal surface dark brown with olivaceous tinge; ascospores 10–13 × 4–5 (–6) μm	***B. ambiens***
–	Mature stromatal surface blackish	**14**
14	Ascospores mostly greater than 16 μm long	**15**
–	Ascospores mostly less than 16 μm long	**16**
15	Perithecia narrow, 0.05–0.1 mm diam; ascospores 20–26 × 10–14(–16) µm	***B. cylindrispora***
–	Perithecia broad, 0.2–0.4 mm diam; ascospores (14– )15–22. 5(–24) × 8–11(–12) µm	***B. capnodes* var. *rumpens***
16	Perithecia less than 0.15 mm diam	**17**
–	Perithecia more than 0.2 mm diam	**18**
17	Ascospores 11–14 × 5.5–7.5 µm, with narrowly or occasionally broadly rounded ends	***B. plana***
–	Ascospores 11–15 × 5.7–7 µm, with broadly rounded ends	***B. petrensis***
18	Germ-slit on both sides of the ascospore, ascospores 13–16 × 5–7 µm	***B. albosticta* var. *orientalis***
–	Germ-slit on one side of the ascospore	**19**
19	Ascospores 8.5–15 × 5–7.5 µm	***B. capnodes***
–	Ascospores 8–11 × 4.4–6 µm	***B. xishuangbannaensis***

## Supplementary Material

XML Treatment for Biscogniauxia diaoluoshanensis

XML Treatment for Biscogniauxia jianfengensis

## Additional information

### Artificial Intelligence (AI) use

The authors accept full responsibility for the content of the manuscript, including the disclosure of any use of AI.

No AI tools were used in the preparation of this manuscript.

### Author ORCIDs

S.-Y. Ma https://orcid.org/0009-0000-2974-6277

S. Liu https://orcid.org/0009-0008-8121-6394

Z. Qu https://orcid.org/0000-0002-0909-0853

H. Jiang https://orcid.org/0009-0006-6430-5788

A.-H. Zhu https://orcid.org/0000-0002-2812-8108

H.-X. Ma https://orcid.org/0000-0001-6699-7454

### Data availability

All of the data that support the findings of this study are available in the main text.
